# Peripapillary choroidal thickness in healthy Chinese subjects

**DOI:** 10.1186/1471-2415-13-23

**Published:** 2013-06-10

**Authors:** Wenbin Huang, Wei Wang, Minwen Zhou, Shida Chen, Xinbo Gao, Qian Fan, Xiaoyan Ding, Xiulan Zhang

**Affiliations:** 1 Zhongshan Ophthalmic Center, State Key Laboratory of Ophthalmology, Sun Yat-Sen University, Guangzhou, People’s Republic of China

**Keywords:** Peripapillary choroidal thickness, Enhanced depth imaging optical coherence tomography, Healthy Chinese subjects

## Abstract

**Background:**

To evaluate the peripapillary choroidal thickness of a healthy Chinese population, and to determine its influencing factors.

**Methods:**

A total of 76 healthy volunteers (76 eyes) without ophthalmic or systemic symptoms were enrolled. Choroidal scans (360-degree 3.4 mm diameter peripapillary circle scans) were obtained for all eyes using enhanced depth imaging spectral-domain optical coherence tomography. Choroid thickness was measured at the temporal, superotemporal, superior, superonasal, nasal, inferonasal, inferior, and inferotemporal segments.

**Results:**

The average peripapillary choroidal thicknesses were 165.03 ± 40.37 μm. Inferonasal, inferior, and inferotemporal thicknesses were significantly thinner than temporal, superotemporal, superior, superonasal, nasal thicknesses (p < 0.05). No statistically significant difference was found among inferonasal, inferior, and inferotemporal thicknesses. The average peripapillary choroidal thickness decreased linearly with age (β = −1.33, 95% CI −1.98, -0.68, P < 0.001). No correlation was noted between average choroidal thickness and other factors (gender, refractive error, axial length, average retinal nerve fiber layer thickness, intraocular pressure, diastolic blood pressure, systolic blood pressure, mean blood pressure, diastolic ocular perfusion pressure, systolic ocular perfusion pressure, and mean ocular perfusion pressure).

**Conclusions:**

The inferonasal, inferior, inferotemporal peripapillary choroidal thicknesses were significantly thinner than temporal, superotemporal, superior, superonasal, and nasal thicknesses. A thinner peripapillary choroid is associated with increasing age.

## Background

The choroid, a highly vascular membrane that covers most of the posterior of the eye between the retina and sclera, provides oxygen and nourishment to the uvea and outer layers of the retina. A functionally normal choroidal vasculature is essential for retinal function; thinning of the choroid and loss of the vascular tissues often leads to photoreceptor damage and vascular dysfunction. The choroid thickness has been found having a vital relationship with the pathophysiology of many conditions, such as age-related macular degeneration (AMD) [1], polypoidal choroidal vasculopathy (PCV) [2], Vogt-Koyanagi-Harada (VKH) [3], glaucoma [4], and others.

A greater understanding of choroidal structure would allow a more accurate evaluation of many posterior segment diseases. Early in the 1970s, scientists attempted to use ultrasound to obtain in vivo measurements of the choroidal thickness [5], but this method lacked reproducibility. However, reliable and accurate measurement of full choroidal thickness is now made possible by the recent development of enhanced depth imaging optical coherence tomography (EDI-OCT) [6-9]. The EDI-OCT provides images of the full-thickness choroid, which enables one to obtain the choroidal thickness by measuring the distance between the retinal pigment epithelium (RPE) and the chorioscleral interface [10]. Current choroidal investigations primarily focus on macular choroidal thickness [6,8,9,11,12], and our research group has also previously investigated the macular choroidal thickness and its profiles in healthy Chinese subjects [13].

Only a few studies have measured peripapillary choroidal thickness [4,14-16]. Given the role of the choroidal vasculature in the blood supply of the anterior optic nerve head, studying on peripapillary choroidal thickness is of great significance. Major ocular pathologies may have associated pathology located in the peripapillary choroidal region; for example, glaucoma eyes have thinner peripapillary choroids [4], and abnormal choroidal blood supply has been suggested as one factor responsible for the occurrence of glaucomatous optic neuropathy in glaucoma patients [17]. Consequently, understanding the normal baseline peripapillary choroidal thickness may aid in elucidating the pathophysiology of these diseases, and might be a useful tool in clinical detection. However, no study has yet measured the peripapillary choroidal thickness in healthy Chinese people. The purpose of this study is to by use EDI-OCT to establish the thickness of the normal peripapillary choroid in a group of healthy Chinese volunteers and to analyze potential influencing factors.

## Methods

All participants in this study received a detailed explanation about the study and signed an informed consent form in accordance with the principles embodied in the Declaration of Helsinki. This study was approved by the Ethical Review Committee of Zhongshan Ophthalmic Center.

In all, 76 healthy Chinese volunteers with no history of eye disorders other than mild to moderate cataracts were enrolled. All subjects were Han nationality Chinese. Exclusion criteria included high myopia or hyperopia (greater than + 6 or −6 diopters of spherical equivalent refractive error (RE)); any retinal or retinal pigment epithelial detachment; any retinal abnormalities such as choroidal neovascularization, asymptomatic pigment epithelial detachment, or whitish myopic atrophy; clinically relevant opacities of the optic media and low-quality images due to unstable fixation, or severe cataract (Patients with mild to moderate cataract might be enrolled in the study, but only high-quality images were included).

All subjects underwent a thorough ophthalmic evaluation, which included slitlamp biomicroscopy, intraocular pressure (IOP) measurement on the day of imaging, fundus examination, a RE examination using an autorefractometer (KR-8900 version 1.07, Topcon Corporation, Tokyo, Japan) and axial length measurements using partial optical coherence inferometry (IOLMaster; Carl Zeiss Meditec, Inc.). Demographic data such as age, gender, systemic disease, systemic antihypertensive medication, diastolic blood pressure (DBP), systolic blood pressure (SBP), mean blood pressure (MBP), diastolic ocular perfusion pressure (DOPP), systolic ocular perfusion pressure (SOPP), and mean ocular perfusion pressure (MOPP) were collected for each subject. MBP, DOPP, SOPP, and MOPP were calculated according to the following formulas [18]: MBP = DBP + 1/3(SBP - DBP), DOPP = DBP - IOP, SOPP = SBP - IOP, and MOPP = MBP - IOP.

**Figure 1:**
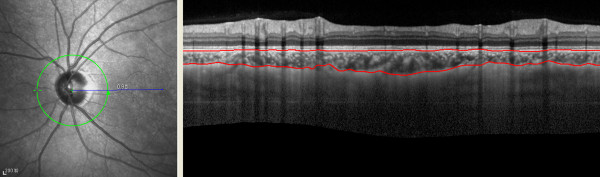
**Images from 360**-**degree 3.4 mm diameter peripapillary circle scans.** Examples of images describing choroidal thickness and demonstrating manual delineation of the choroidal vasculature lying between the outer border of the retinal pigment epithelium (RPE) and the inner surface of the sclera.

All subjects were examined with undilated pupils using an EDI system of multimodality diagnostic imaging (wavelength: 870 nm; scan pattern: enhanced depth imaging; Spectralis HRA + OCT; Heidelberg Engineering, Heidelberg, Germany). The image was averaged for 100 scans using the automatic averaging and eye tracking features. For measurements of peripapillary choroidal thickness, a 360-degree 3.4 mm diameter peripapillary circle scan was performed using the standard protocol for retinal nerve fiber layer (RNFL) assessment, as previously described [16]. The RNFL thickness was also determined using the same peripapillary circle scan. Only the right eye of each study participant was assessed. The resultant images were viewed and measured with the supplied Heidelberg Eye Explorer software (version 1.5.12.0; Heidelberg Engineering). Keratometry readings and the most recent refraction were entered into the software program to estimate optical magnification and, therefore, to allow for more accurate comparisons across individuals. The choroid thickness was measured manually from the outer portion of the hyperreflective line corresponding to the RPE to the inner surface of the sclera (Figure 1). Choroid thickness was measured at the temporal, superotemporal, superior, superonasal, nasal, inferonasal, inferior, and inferotemporal segments. The choroid was measured by two independent graders. If the thickness difference measurements of the two examiners exceeded 15% of the mean of the two values, there was open adjudication with the senior author and then averaged for analysis.

The data were processed and statistically analyzed using a commercial analytical software program (SPSS 17.0; SPSS, Inc., Chicago, IL). One-way analysis of variance was used to compare choroidal thickness at different segments. Simple linear regression and multiple linear regression were calculated for variations in average peripapillary choroidal thickness relative to age, gender, RE, axial length, average RNFL thickness, IOP, DBP, SBP, MBP, DOPP, SOPP, and MOPP. All statistical tests were two-sided with a 0.05 level of significance.

## Results

### Demographic data

Relevant characteristics of the included subjects are given in Table 1. In total, 39 men and 37 women (mean age, 56.95 ± 12.99 years; range, 18–78 years) were enrolled. The mean spherical equivalent was 0.31 ± 1.12 D (range, +3.0 to −3.0 D). The mean axial length was 23.20 ± 0.81 mm. The average RNFL thickness was 110.54 ± 25.53 μm. The mean IOP was 15.09 ± 3.69 mmHg. The mean DBP was 75.00 ± 8.55 mmHg. The mean SBP was 123.26 ± 17.26 mmHg. The mean MBP was 91.09 ± 9.59 mmHg. The mean DOPP was 59.91 ± 8.81 mmHg. The mean SOPP was 108.18 ± 16.67 mmHg. The mean MOPP was 76.00 ± 9.40 mmHg.

**Table 1:** Clinical characteristics of the study subjects

	**Study subjects**	**SD**
No. of patients (No. of eyes)	76 (76)	-
Age, y	56.95	12.99
Gender (male/female)	39/37	-
Spherical equivalent, D	0.31	1.12
Axial length, mm	23.20	0.81
average RNFL thickness, μm	110.54	25.53
IOP at imaging, mmHg	15.09	3.69
DBP, mmHg	75.00	8.55
SBP, mmHg	123.26	17.26
MBP, mmHg	91.09	9.59
DOPP, mmHg	59.91	8.81
SOPP, mmHg	108.18	16.67
MOPP, mmHg	76.00	9.40

Abbreviations: *D* diopter, *RNFL* retinal nerve fiber layer, *IOP* intraocular pressure, *DBP* diastolic blood pressure, *SBP* systolic blood pressure, *MBP* mean blood pressure, *DOPP* diastolic ocular perfusion pressure, *SOPP* systolic ocular perfusion pressure; *MOPP* mean ocular perfusion pressure, *SD* standard deviation.

### Peripapillary choroidal thickness measurements

**Figure 2:**
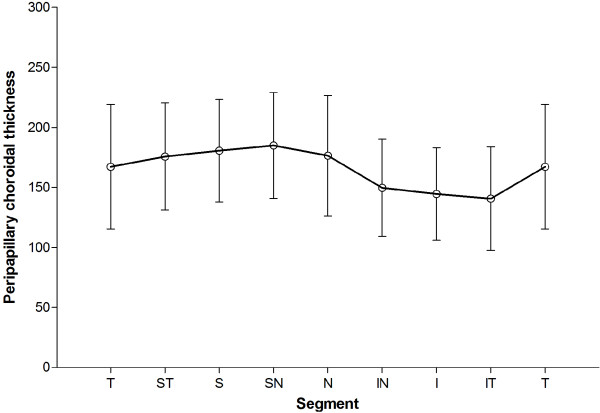
**Variation trend of peripapillary choroidal thickness.** The superonasal segment had the thickest choroidal thickness, while the inferotemporal segment had the thinnest thickness.

The average peripapillary choroidal thicknesses were 165.03 ± 40.37 μm with 95% confidence interval (CI) 155.80–174.25 μm in 76 subjects. The choroidal thickness at different locations is shown in Table 2. The temporal, superotemporal, superior, superonasal, and nasal segment had thicker choroidal thicknesses at 167.26 ± 51.86 μm, 175.70 ± 44.55 μm, 180.65 ± 42.72 μm, 185.05 ± 44.14 μm, and 176.42 ± 50.16 μm, respectively. In contrast, the inferonasal, inferior, and inferotemporal segments had thinner choroidal thickness at 149.64 ± 40.53 μm, 144.54 ± 38.59 μm, and 140.70 ± 43.15 μm, respectively. Post hoc analysis utilizing least significant difference (LSD) t-Text demonstrated that the inferonasal, inferior, and inferotemporal thicknesses were significantly thinner than the temporal, superotemporal, superior, superonasal, nasal thicknesses (p < 0.05, Table 3). However, no statistically significant difference was noted among the inferonasal, inferior, and inferotemporal thicknesses. None of other segments (superotemporal, superior, superonasal, and nasal) demonstrated any significant differences with each other (Table 3). The variation trend of peripapillary choroidal thickness is illustrated in Figure 2.

**Table 2:** **Average choroidal thickness and 95% CI at different segments with 360**-**degree 3.4 mm diameter peripapillary circle scans**

**Segment**	**Average choroidal thickness (μm)**	**SD**	**95% CI**	
			**Lower bound**	**Upper bound**
T segment	167.26	51.86	155.41	179.11
ST segment	175.70	44.55	165.52	185.88
S segment	180.65	42.72	170.89	190.41
SN segment	185.05	44.14	174.97	195.14
N segment	176.42	50.16	164.96	187.88
IN segment	149.64	40.53	140.38	158.91
I segment	144.54	38.59	135.72	153.36
IT segment	140.70	43.15	130.84	150.56
Average	165.03	40.37	155.80	174.25

Abbreviations: *T* temporal, *ST* Superotemporal, *S* Superior, *SN* Superonasal, *N* nasal, *IN* Inferonasal, *I* Inferior, *IT* Inferotemporal, *SD* standard deviation, *CI* confidence interval.

**Table 3:** **Post hoc analysis of the peripapillary choroidal thickness at eight segments using least significant difference (LSD) t**-**Text**

**Segment**	**T**	**ST**	**S**	**SN**	**N**	**IN**	**I**	**IT**
T	-	-	-	-	-	-	-	-
ST	0.245	-	-	-	-	-	-	-
S	0.065	0.494	-	-	-	-	-	-
SN	**0**.**014**	0.197	0.544	-	-	-	-	-
N	0.207	0.920	0.560	0.234	-	-	-	-
IN	**0**.**015**	<**0**.**001**	<**0**.**001**	<**0**.**001**	<**0**.**001**	-	-	-
I	**0**.**002**	<**0**.**001**	<**0**.**001**	<**0**.**001**	<**0**.**001**	0.481	-	-
IT	<**0**.**001**	<**0**.**001**	<**0**.**001**	<**0**.**001**	<**0**.**001**	0.217	0.596	-

Bold values represent significance. Abbreviations: *T* temporal, *ST* Superotemporal, *S* Superior, *SN* Superonasal, *N* nasal, *IN* Inferonasal, *I* Inferior, *IT* Inferotemporal.

### Regression analysis

**Figure 3:**
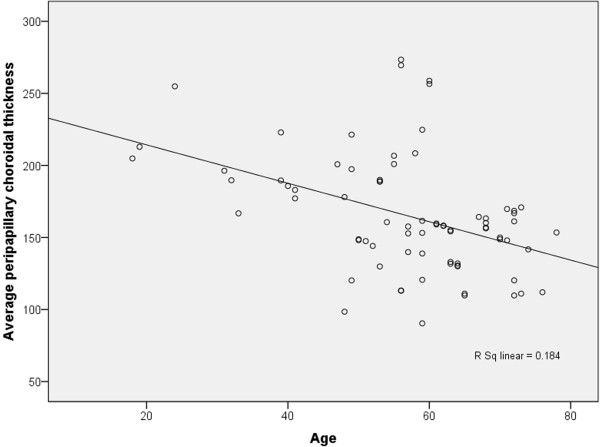
**Scatterplot of average peripapillary choroidal thickness and age in healthy subjects.** Average peripapillary choroidal thickness is negatively correlated with age. r = 0.429, R^2^ = 0.184.

Simple linear regression was used to determine any influencing factors associated with average peripapillary choroidal thickness (Table 4). Average choroidal thickness in healthy controls decreased linearly with age (β = −1.33, 95% CI −1.98, -0.68, P < 0.001). A scatterplot of the simple linear regression analysis between average peripapillary choroidal thickness and age is shown in Figure 3. However, other factors (gender, RE, axial length, average RNFL thickness, IOP, DBP, SBP, MBP, DOPP, SOPP, and MOPP) did not relate significantly to average peripapillary choroidal thickness. Stepwise multiple regression analysis only found age was related significantly with average peripapillary choroidal thickness (P < 0.001).

**Table 4:** Average peripapillary choroidal thickness and univariable associations

	**Beta [95% CI]**	**P value**
Age, y	−1.33 [−1.98, -0.68]	<**0**.**001**
Male gender ( vs. female)	13.78 [−4.52, 32.09]	0.138
Spherical equivalent, D	−3.69 [−11.99, 4.61]	0.379
Axial length, mm	2.22 [−9.36, 13.79]	0.704
RNFL thickness, μm	0.18 [−0.18, 0.55]	0.322
IOP at imaging, mmHg	1.77 [−0.73, 4.27]	0.163
DBP, mmHg	0.49 [−0.60, 1.57]	0.376
SBP, mmHg	0.00 [−0.54, 0.54]	0.998
MBP, mmHg	0.26 [−0.71, 1.23]	0.598
DOPP, mmHg	0.15 [−0.91, 1.21]	0.782
SOPP, mmHg	−0.09 [−0.65, 0.47]	0.761
MOPP, mmHg	0.00 [−1.00, 0.99]	0.994

Bold values represent significance. Abbreviations: *D* diopter, *RNFL* retinal nerve fiber layer, *IOP* intraocular pressure, *DBP* diastolic blood pressure, *SBP* systolic blood pressure, *MBP* mean blood pressure, *DOPP* diastolic ocular perfusion pressure, *SOPP* systolic ocular perfusion pressure, *MOPP* mean ocular perfusion pressure.

## Discussion

The peripapillary choroid, which affects the optic nerve and retina, plays a vital role in the pathophysiology of many diseases, including glaucoma [4] and high myopia [19]. Qualitative and quantitative analysis of peripapillary choroidal thickness may help us to evaluate its physiopathological changes, to track disease progression, and potentially to measure responses to therapy. Despite its importance, the choroidal circulation located beneath the retina and the retinal pigment epithelium is difficult to study without improvement of OCT technology. To the best of our knowledge, the present study is the first to obtain detailed information on the peripapillary choroid of healthy Chinese subjects using EDI-OCT.

Of the few published studies that have characterized the peripapillary choroidal thickness in normal subjects, the study by Roberts and colleagues [4] showed a mean peripapillary choroidal thickness of 154 μm in 76 healthy Canadian subjects (mean age, 56.1 years), determined using SD-OCT (12° circular scan protocol centered on optic nerve head). Ho and colleagues [15] mapped the peripapillary choroid in 36 normal subjects (mean age, 48 years) using the Cirrus HD-OCT (a horizontal and a vertical scan). Overall, they showed that the peripapillary choroid in the inferior quadrant was significantly thinner than in the other quadrants. At 1 mm away from optic nerve, the temporal, superior, nasal, and inferior choroid values were 204, 236, 234, and 151 μm, respectively. This normal choroidal thickness value at 1 mm away from optic nerve corresponds to a distance of about 1.7 mm away from the center of optic nerve head since the radius of the optic disk is about 0.75 mm. In the present study, our 3.4 mm diameter SD-OCT circle scans measured choroidal thickness 1.7 mm away from the center of optic nerve head. We showed that choroidal thickness in our 76 healthy subjects, with a mean age of 56.95 years, was 167.26 ± 51.86 μm temporally, 175.70 ± 44.55 μm superotemporally, 180.65 ± 42.72 μm superiorly, 185.05 ± 44.14 μm superonasally, 176.42 ± 50.19 μm nasally, 149.64 ± 40.53 μm inferonasally, 144.54 ± 38.59 μm inferiorly, and 140.70 ± 43.15 μm inferotemporally; and the average choroidal thickness was 165.03 ± 40.37 μm. These values were somewhat different from the values reported by Roberts and Ho. The disparities may arise from differences in the measuring software or the OCT light source, differences in ethnicity, or differences in patient profiles. Moreover, date from Ho and colleagues were collected on a point-to-point basis, and no continuous measurement of the choroidal thickness was made around the optic disk. The 360-degree peripapillary scan technique presented here provided continuous peripapillary choroidal thickness measurements. However, we consistently showed that the inferior region was significantly thinner when compared to other regions. Using EDI-OCT, Tanabe and colleagues also found that the choroid is thinner in the inferior region of optic disks of eyes of normal Japanese persons [14].

The reason why the peripapillary choroid is thinner in the inferior region than in the other regions is not clear. One possibility is that this regional difference is a result of the developmental pattern of the eye. Evidence from embryology indicates that the optic fissure is located in the inferior aspect of the optic cup, which is the last part of the globe to close during eye formation [20]. A thinner choroid may increase vascular resistance, resulting in decreased blood flow in the choriocapillaris, which may be more susceptible to hypoxia or to elevated IOP. For example, the thinner choroid in the inferior region may make the inferior optic disk more vulnerable to choroidal circulation flow changes. This concept is supported by the observation that the superior hemifield is affected more often and more severely than the inferior hemi field in glaucomatous eyes [14,21].

Earlier studies have reported negative correlations between peripapillary choroidal thickness and age [4,21]. Regression analysis suggested an approximate 11 μm decrease in peripapillary choroidal thickness per decade by Roberts and colleagues [4]. In the present study, we found a similar decrease of 13μm over the same period of time. The RE and axial length were found to correlate with macular choroidal thickness in previous studies of us and others [13,22], but no correlation was noted between peripapillary choroidal thickness and RE or axial in this present study. One possible reason is that the demographic data of the enrolled subjects is different, and that the variation range of peripapillary choroidal thickness affected by the axial length is smaller than macular choroidal thickness since the average peripapillary choroidal thickness is much thinner than the macular choroidal thickness.

Because the choroid is a highly vascular structure, the thickness may vary with the IOP and perfusion pressure. In a recent study, Maul et al. found that peripapillary choroidal thickness was significantly associated with DBP and DOPP in glaucomatous eyes [18]. However, in our present study, no significant associations were detected between the peripapillary choroidal thickness and the IOP, DBP, SBP, MBP, DOPP, SOPP, and MOPP. This may be because the auto regulation of choroidal blood flow only causes slight changes in choroidal thickness within the normal range of IOP and ocular perfusion pressure in healthy subjects.

There are some potential limitations in our study. First, the current operating software of the Hei delberg Spectralis OCT does not provide automatic segmentation of the choroid. It is necessary to assess the reproducibility for measurement of peripapillary choroidal thickness. However, in a recent study Ehrilich and colleagues reported that Lin’s concordance correlation coefficient (CCC) for peripapillary choroidal thickness measurement was 0.93 (P < 0.001) [16]. The measurements were extremely reproducible. Second, in the present study, choroidal thickness was examined only in the right eye of each subject, so that inter-eye differences and their associations with inter-eye differences of other parameters could not be assessed. Third, there seems to be significant circadian (diurnal) variation in choroidal thickness measurements by OCT [10,23]. The participants in our study underwent the OCT examinations in the morning.

## Conclusions

In summary, this was the first study to investigate the peripapillary choroid of healthy Chinese subjects. We showed that the inferonasal, inferior, and inferotemporal peripapillary choroidal thicknesses in healthy subjects were significantly thinner than the temporal, superotemporal, superior, superonasal, and nasal thicknesses. A thinner peripapillary choroid is associated with increasing age. More studies are needed to study the peripapillary choroid profiles and its influencing factors.

## Competing interests

The authors declare that they have no competing interests.

## Authors’ contributions

All authors conceived of and designed the experimental protocol. WH and WW collected the data. All authors were involved in the analysis. WH wrote the first draft of the manuscript. WH, WW and XZ reviewed and revised the manuscript and produced the final version. All authors read and approved the final manuscript.

## Pre-publication history

The pre-publication history for this paper can be accessed here:

http://www.biomedcentral.com/1471-2415/13/23/prepub
